# Evaluation eines nutzerorientierten eLearning-Angebots für die Augenheilkunde

**DOI:** 10.1007/s00347-020-01306-z

**Published:** 2021-01-06

**Authors:** Andreas Müller, Franziska Schmidt, Norbert Pfeiffer, Anita Brill, Verena Prokosch

**Affiliations:** 1 Augenklinik und Poliklinik, Universitätsmedizin Mainz, Johannes Gutenberg-Universität Mainz, Langenbeckstr. 1, 55131 Mainz, Deutschland; 2 Zentrum für Qualitätssicherung und -entwicklung, Johannes Gutenberg Universität Mainz, Mainz, Deutschland; 3 Zentrum für Augenheilkunde, Uniklinik Köln, Universität zu Köln, Köln, Deutschland

**Keywords:** Studium, Augenheilkunde, Lernverhalten, E‑Learning, Vernetzung, Medical school, Ophthalmology, E‑Learning, Learning habit, Networking

## Abstract

**Hintergrund:**

Digitales Lernen wird in humanmedizinischen Studiengängen meist mittels Learning-Management-Plattformen wie „Ilias“ umgesetzt. Oft werden Vorlesungsfolien und Organisatorisches hierbei online gestellt. Allerdings nutzen Studierende auch externe Quellen und Lernformate in ihrem Lernprozess. Wir stellen hier die Neugestaltung unseres digitalen Lernangebotes und dessen Beurteilung durch die Studenten vor.

**Ziel der Arbeit:**

Evaluation einer eLearning-Plattform für Studierende der Humanmedizin, welche übliches Lernverhalten berücksichtigt.

**Material und Methoden:**

Die Plattform sollte, geordnet nach Augenstrukturen, eine Auswahl an in- und externen Lernmaterialien verknüpfen. Zu jedem Thema erstellten wir ein Skript zu den jeweiligen zentralen Inhalten, Fallbeispiele, Vorlesungsfolien, Übungsfragen und Kapitel von Lehrbüchern sowie die entsprechende Amboss-Lernkarte (Amboss GmbH, Berlin, Deutschland). Am Semesterende erfolgte eine Evaluation der Plattform. In einem Fragebogen wurden verschiedene Aussagen auf Ordinalskalen von Studierenden bewertet.

**Ergebnisse:**

Die Plattform wurde mit 1,47 ± 0,54 (Mittelwert ± Standardabweichung) bewertet (*n* = 107; Schulnote 1 = sehr gut, 6 = ungenügend). Sie wurde mit 1,62 ± 0,77 als sehr hilfreich für das eigene Lernen empfunden (1 = sehr hilfreich, 7 = gar nicht hilfreich). Die Strukturierung der internen und externen Lernmaterialien wurde als sehr gut mit 1,44 ± 0,66 bewertet (1 = sehr gut, 7 = sehr schlecht). Im Median wurde eine Nutzungsdauer von 1–5 h angegeben (Ordinalskala <1, 1–5, 6–10, 15–20, >20 h).

**Diskussion:**

Es erscheint sinnvoll, häufig genutzte externe Lernmaterialien gezielt mit der eigenen curricularen Struktur und ihren Inhalten zu verknüpfen und digital auf einer Plattform zur Verfügung zu stellen, um eine zeitgemäße Basis für Wissenserwerb für Studierende in der Augenheilkunde zu schaffen.

## Abstract (en)

**Background:**

In medical studies digital learning is often achieved by the use of learning management platforms, such as Ilias. Lecture presentations and organizational documents are typical contents. Students use multiple, mostly external learning options for acquisition of knowledge and competences. We present our updated ophthalmology e‑learning environment for medical students and its evaluation.

**Objective:**

Evaluation of an ophthalmology e‑learning platform for medical students, which considers prevalent learning habits.

**Material and methods:**

The platform should provide and link a selection of internal and external learning resources following anatomical structures of the eye. For every subject area the platform provides a text with essential matters, clinical cases, lecture presentations, multiple choice questions for practice, links to corresponding chapters of a textbook and the appropriate AMBOSS learning cards (Amboss GmbH, Berlin, Germany).

At the end of the semester an evaluation of the platform was carried out. Different statements were rated by the students on ordinal scales and analyzed.

**Results:**

The platform was rated with 1.47 ± 0.54 (mean ± standard deviation; *n* = 107) on a German school grade scale (1 = best, 6 = worst). It was perceived as helpful for the individual learning with 1.62 ± 0.77 (1 = very helpful, 7 = not helpful at all). The structuring of the internal and external learning resources was rated as very good 1.44 ± 0.66 (1 = very good, 7 = very bad). The median for subjective amount of usage was 1–5 h (ordinal scale <1, 1–5, 6–10, 15–20, >20 h).

**Conclusion:**

It appears to be meaningful to specifically link external learning resources corresponding to the own curricular structure in order to provide medical students with a modern basis for learning in ophthalmology.

An einem digitalen Lernangebot für Medizinstudierende führt heutzutage kein Weg mehr vorbei. Als visuelles Fach sind die Möglichkeiten der Abbildung von Lerninhalten der Augenheilkunde vielfältig. Jedoch gilt es, bei der Präsentation digitaler Lehrinhalte das Lernverhalten der Studierenden zu beachten. Die Nutzung von Lernmaterialien aus mehreren Quellen ist Praxis. Dies bezogen wir bei der Neugestaltung unseres eLearning-Angebotes im Wintersemester 2019/20 ein und ließen die neue Plattform durch die Studierenden im Kurs der Augenheilkunde evaluieren.

## Einleitung

Die beste Art und Weise von Wissenserwerb ist individuell unterschiedlich. Mittlerweile stehen durch die digitalen Medien und das Internet mehr Bücher, Skripte, Websites, Bilderdatenbanken, Videos und Lernmaterialien als jemals zuvor zur Verfügung. Im universitären Setting möchten Dozierende aber auch eigene Schwerpunkte vermitteln.

Das gegenwärtige Lernverhalten ist von der Nutzung unterschiedlicher Lernmaterialien geprägt [3]. Während das gedruckte Lehrbuch weiterhin Lesende findet, liegen diese auch wie die meisten Lernmaterialien digital vor. Bei der Schaffung eigener digitaler Lernmaterialien und insbesondere Lernplattformen gilt es daher, nicht nur klar Zweck und Methode des Lernangebotes zu formulieren, sondern auch das Lernverhalten der Studierenden zu berücksichtigen [2, 6].

Um die Studierenden in ihrem Kompetenzerwerb zu unterstützen, konzipierten wir die Neugestaltung unserer Lernplattform als Knotenpunkt zwischen internen und externen Lernmaterialien mit einer Mischung aus frontalen und interaktiven Angeboten sowie Zugriff auf alle Detailtiefen von Skripten über Amboss-Lernkarten (Amboss GmbH, Berlin, Deutschland) bis hin zu Lehrbüchern. Wir führten gemeinsam mit dem Zentrum für Qualitätssicherung der Johannes-Gutenberg-Universität eine umfangreiche Evaluation der eLearning-Plattform durch und erhoben objektive Parameter des Nutzungsumfangs.

## Methodik

Die eLearning-Plattform war formativer – also nicht für das Bestehen oder die Benotung des Kurses relevanter – Bestandteil des Curriculums für das Praktikum der Augenheilkunde, welches an der Universitätsmedizin Mainz im sechsten Semester stattfindet. Bestandteile des Praktikums (bis einschließlich des Wintersemesters 19/20) waren eine wöchentlich stattfindende Vorlesung, ein Praktikum welches innerhalb 1 Woche in Kleingruppen absolviert wurde (Einführung, Übung an der Spaltlampe, Anamnese & Untersuchung eigener Patienten, Hospitation in Sprechstunden und im Operationssaal) sowie eine schriftliche Klausur mit Prüfungsfragen zu den wesentlichen Inhalten der Augenheilkunde, welche Ärzten jeder Fachrichtung geläufig sein sollten. Die Online-Plattform stand den Studierenden ab dem ersten Vorlesungstermin zur Verfügung. Einen allgemeinen Untersuchungskurs Augenheilkunde absolvierten die Studierenden bereits im 5. Semester. Parallel zu dem Praktikum der Augenheilkunde im 6. Semester finden curricular zusätzlich Kurse der Pharmakologie, HNO, klinischen Chemie, Inneren Medizin, Mikrobiologie/Hygiene, Anästhesie sowie Medizinethik statt.

Die eLearning-Plattform wurde mittels „exelearning“ (V. 2.4 open-source Authoring-Software, exelearning.net) umgesetzt und als HTML-Website im ILIAS Learning Management System (LMS) eingebunden (V. 5.4, ILIAS open source e‑Learning e. V., Köln).

**Figure 1:**
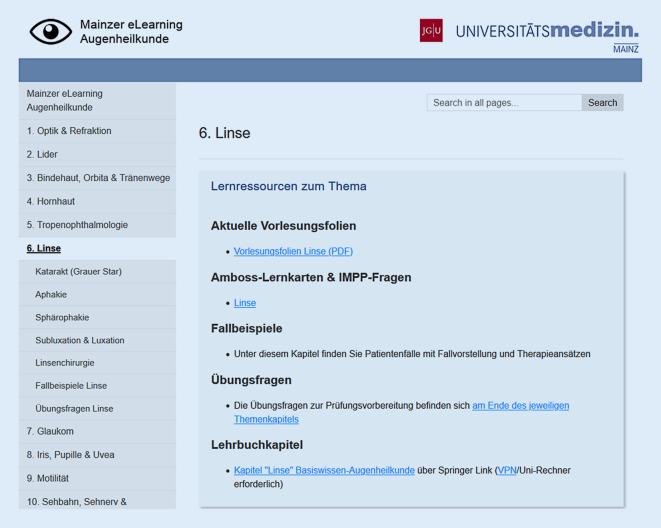

Die Plattform stellt, geordnet nach Themen analog zu den Themen der Vorlesungsreihe (z. B. „Hornhaut“, s. Tab. 1), eine Übersichtsseite bereit mit relevanten eigenen und externen Lernmaterialien (aktuelle Vorlesungsfolien, zugehörige Amboss-Lernkarte, eigene Fallbeispiele, Multiple-Choice Übungsfragen und das zugehörige Lehrbuchkapitel) sowie in untergeordneten Seiten ein Skript zu ausgewählten Aspekten des jeweiligen Themas (Abb. 1). Die zugehörigen Lehrbuchkapitel können über entsprechende Universitätslizenzen unmittelbar online beim Verlag aufgerufen werden, solange die Lernplattform entweder von Uni-Rechnern oder per VPN-Client am eigenen Rechner aufgerufen wird.

**Table 1:** 

Für jedes Thema direkter Link zu: Aktuellen Vorlesungsfolien, Amboss-Lernkarten (Amboss GmbH, Berlin, Deutschland), Fallbeispielen, Übungsfragen, Lehrbuchkapiteln										
Optik & Refraktion	Lider	Bindehaut, Orbita & Tränenwege	Hornhaut	Tropenophthalmologie	Linse	Glaukom	Iris, Pupille & Uvea	Motilität	Sehbahn, Sehnerv & Traumatologie	Netzhaut
Brechung, Myopie und Hyperopie	Allergische Lidveränderungen	Bindehaut	Entzündungen der Hornhaut	Katarakt als Erblindungsursache	Katarakt (grauer Star)	Primärglaukome	Iris & Pupille	Strabismus concomitans	Sehbahn & Sehnerv	Amotio retinae
Refraktive Chirurgie	Entzündliche Liderkrankungen	Orbita und Tränendrüse	Degenerationen der Hornhaut	Trachom	Aphakie	Sekundärglaukome	Uvea	Lähmungsschielen	Gesichtsfelddefekte	Diabetische Retinopathie
	Fehlstellungen und Anomalien	Abführende Tränenwege und Tränenfilm	Dystrophien der Hornhaut	Lepra	Sphärophakie	Therapie	Glaskörper	Amblyopie	Traumatologie	Hereditäre Netzhauterkrankungen
	Benigne Tumoren		Traumata der Hornhaut	Onchozerkose	Subluxation & Luxation	Klinische Untersuchung & Glaukomdiagnostik				Hypertensive Retinopathie
	Maligne Tumoren		Keratokonus		Linsenchirurgie					Pathologien der Makula
			Hornhautchirurgie							Retinale Gefäßverschlüsse
										Tumoren der Retina

**Figure 2:**
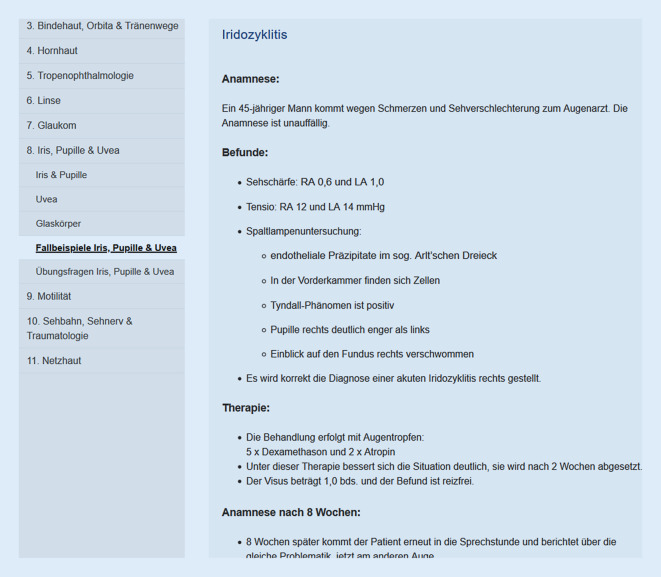

Die Fallbeispiele sind nach dem klinischen Vorgehen (Anamnese, Untersuchung, Diagnose, Therapie) beschriebene Patientenverläufe, welche die praktische Relevanz der klinisch-theoretischen Inhalte veranschaulichen sollen (Abb. 2).

**Figure 3:**
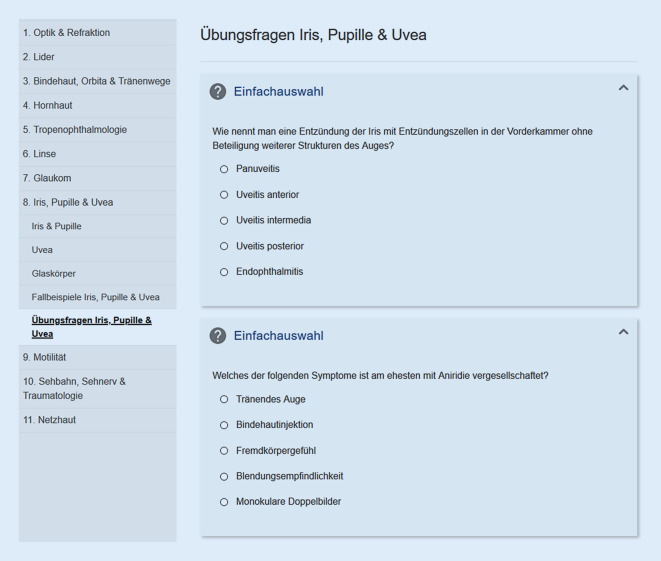

Ein Multiple-Choice-Fragentool ist inhaltlich mit in früheren Semestern gestellten Klausurfragen ausgestattet (Abb. 3).

Es nahmen 157 Studierende im 6. Fachsemester an der Evaluation nach Teilnahme an Vorlesung und Praktikum der Augenheilkunde der Poliklinik und Augenklinik der Universitätsmedizin Mainz im Februar 2020 teil. Die Studierenden wurden im Laufe des Semesters bei Einführungsveranstaltung, per E‑Mail und in 2 weiteren Vorlesungen auf das eLearning-Angebot hingewiesen.

Die Studierenden wurden aufgefordert, nach Abschluss der Klausur einen Evaluationsbogen auszufüllen, welcher in Zusammenarbeit mit dem Zentrum für Qualitätssicherung und -entwicklung (ZQ) der Johannes-Gutenberg-Universität Mainz gestaltet wurde. Auf diesem wurden zur eLearning-Plattform sowie zur gesamten Veranstaltung Schulnoten vergeben und auf Likert-Skalen Aussagen zur Veranstaltung und der Einschätzung des individuellen Interesses an der Augenheilkunde bewertet. Weiterhin konnten Freitextantworten verfasst werden. Das ZQ wertete die Fragebögen anschließend aus.

Weiterhin wurden über das LMS die Zugriffszahlen der Studierenden dokumentiert, welche anonymisiert vom ZQ mit den Evaluationsergebnissen zurückgeleitet wurden. Wir werteten diese Daten zur Objektivierung der tatsächlichen Nutzung der Plattform aus. In einer weiterführenden separaten Auswertung schlossen wir NutzerInnen, welche die Plattform lediglich einmal aufriefen, aus, um das Nutzungsverhalten regelmäßiger Nutzer in den Ergebnissen besser abbilden zu können.

## Ergebnisse

Es wurden 157 Evaluationsbögen ausgewertet. Für die jeweiligen Aussagen bzw. Fragen konnten zwischen *n* = 59 und *n* = 157 Fragebögen einbezogen werden. Die Freitextkommentare wurden zur internen Evaluation des Veranstaltungserfolges und der eLearning-Plattform herangezogen und spiegelten die quantitativen Evaluationsergebnisse gut wider.

Übergreifend wurde der Unterricht der Augenklinik im Mittel mit einer Schulnote von 1,78 ± 0,82 (Mittelwert [MW] ± Standardabweichung [SD]; 1 = sehr gut, 6 = ungenügend) bewertet (*n* = 148). Das Interesse am Fach Augenheilkunde stieg von 4,26 ± 1,55 (MW ± SD; *n* = 150; 1 = sehr großes Interesse, 7 = sehr geringes Interesse) auf 3,07 ± 1,32 (MW ± SD; *n* = 149) an.

### Evaluation der eLearning-Plattform

Es gaben 75,9 % der Studierenden in der Evaluation an, die Lernplattform zu kennen (*n* = 141). Die eLearning-Plattform wurde im Mittel mit einer Schulnote von 1,47 ± 0,54 (MW ± SD; 1 = sehr gut, 6 = ungenügend) bewertet (*n* = 107). Sie wurde mit 1,62 ± 0,77 als sehr hilfreich für das eigene Lernen empfunden (1 = sehr hilfreich, 7 = gar nicht hilfreich; *n* = 97). Der Aufbau nach Themen wurde als sehr gut mit 1,39 ± 0,64 bewertet (1 = sehr gut, 7 = sehr schlecht; *n* = 96). Die Auswahl der Fallbeispiele wurde mit 1,78 ± 0,86 als sehr gut empfunden (1 = sehr gut, 7 = sehr schlecht; *n* = 85). Die Auswahl der Übungsfragen wurde mit 1,77 ± 1,09 ebenfalls als sehr gut empfunden (1 = sehr gut, 7 = sehr schlecht; *n* = 95).

Das Angebot der Unterstruktur von eigenen und externen Lernmaterialien wurde als sehr gut mit 1,44 ± 0,66 bewertet (1 = sehr gut, 7 = sehr schlecht; *n* = 96).

### Untersuchung des Nutzungsverhaltens

**Figure 4:**
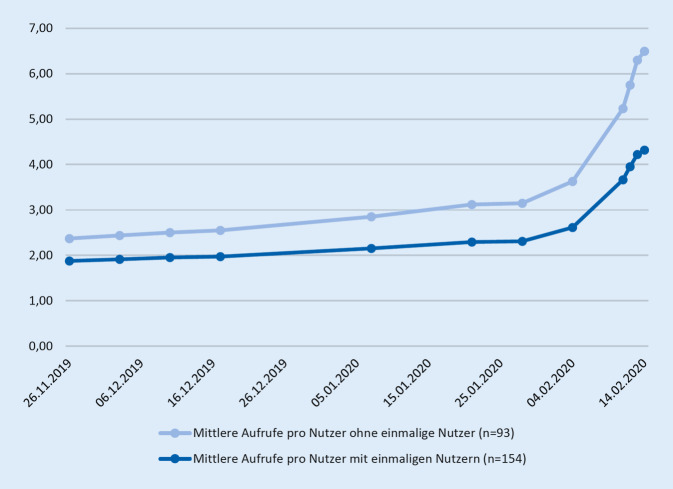

**Figure 5:**
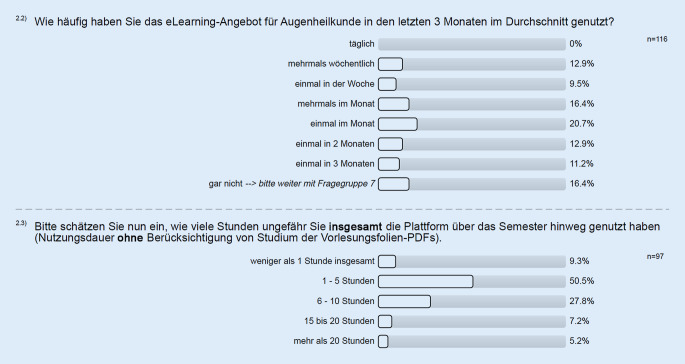

Die eLearning-Plattform wurde von 154 Studierenden insgesamt für 666 Sessions (Zugriffe von ILIAS ausgehend für jeweils eine zusammenhängende Nutzung bis Schließen des Browser-Fensters) aufgerufen. Im Median führte jeder Studierende 2 Sessions (MW 4,32; Min. 1, Max. 37) durch. Unter Ausschluss von Nutzern, die die Plattform nur einmalig aufriefen, führten 93 Studierende insgesamt 605 Sessions durch, hierbei im Median 5 Sessions (MW 6,51; Min. 2, Max. 37). Der Schwerpunkt der Nutzung lag in den 2 Wochen vor dem Klausurtermin (Abb. 4). In einer Selbsteinschätzung der Studierenden wurde die Plattform im Median 1–5 h genutzt. Weitere 27,8 % schätzten die Nutzung mit 6–10 h ein; 12,4 % nutzten die Plattform mehr als 15 h (Abb. 5).

## Diskussion

Bei Konzeption und Gestaltung einer digitalen Online-Lernplattform begeben sich Lehrende schnell in Konkurrenz zu gewerblichen Angeboten wie beispielsweise Amboss (Amboss GmbH, Berlin, Deutschland). Das digitale Angebot eigener Lerninhalte kann schnell redundant gegenüber diesen gewerblichen Angeboten wirken. Wir sehen daher die Auflösung dieser Situation nicht darin, konkurrierende Angebote zu schaffen. Vielmehr sehen wir große Chancen in der Vernetzung der Angebote verschiedener Anbieter und Quellen. Dies ermöglicht den Lernenden, die Vorteile sowohl von in- als auch von externen Materialien zu nutzen, ohne Zeit mit der Recherche, Sammlung und dem Aufruf solcher zu verlieren [6]. Ebenso kann vonseiten der Lehrenden auf diese Weise eine Auswahl qualitativ hochwertiger, verlässlicher Quellen angeboten werden. Hierbei ist es sinnvoll, auch interne Lernmaterialien anzubieten, welche extern nicht zur Verfügung stehen. Beispiele hierfür sind Fallstudien, interaktive Patientenfälle und Übungsfragen, welche ähnliche Schwerpunkte wie z. B. eine hausinterne Klausur abfragen. Die Übersichtlichkeit der Lernmaterialien mit unterschiedlichem Detailgrad (Vorlesungsfolien, Skript, Amboss, Lehrbuch) wurde von Studierenden ebenfalls positiv zurückgemeldet. Das Konzept einer eLearning-Plattform, welche multiple interne und externe Lernmaterialien eng verknüpft, wurde sehr gut angenommen und scheint erfreulicherweise die Lerngewohnheiten der Studierenden zu unterstützen.

Zur qualitativen Bewertung erscheint uns jedoch auch die quantitative Einschätzung von Nutzungsdauer und Zugriffen wichtig. Es zeigte sich, dass in etwa die Hälfte der Studierenden des Kurses die eLearning-Plattform als formatives Lernangebot freiwillig regelmäßig (häufiger als einmalig) nutzte. Überraschend war, dass zum Zeitpunkt der Evaluation trotz umfangreicher Bewerbung der Plattform diese lediglich 75,9 % der Klausur‑/Evaluationsteilnehmer bekannt war. Dies war nur zum Teil durch Klausurwiederholer aus einem vorigen Semester zu erklären. Bei der Einrichtung von eLearning-Angeboten ist daher ratsam, eine zusätzliche Verknüpfung mit Inhalten zu schaffen, welche die Studierenden ohnehin nutzen müssen (z. B. organisatorische Inhalte wie Gruppeneinteilungen oder Terminlisten von Präsenzveranstaltungen). So könnte die Sichtbarkeit einer solchen Plattform mutmaßlich gesteigert werden.

Der Schwerpunkt der Nutzung unseres Angebotes liegt unmittelbar vor der Klausur. Die Annahme, dass viele Studierende erst unmittelbar vor der Klausur lernen, wird häufig getroffen. Unsere Analyse bestätigt dieses vermutete Lernverhalten einer Semesterkohorte in Bezug auf unsere eLearning-Plattform bei einer einmaligen Prüfung am Ende eines Semesters. Dies sehen wir als Abbildung eines Leitsatzes der Lehrforschung: „assessment drives learning“ [5, 7]. Unsere Daten zeigen, dass sich dies nicht nur auf Inhalte, sondern auch auf den Zeitpunkt des Lernens bezieht.

Die meisten Studierenden, welche die Plattform nutzen, verbrachten zwischen 1 und 10 h auf dieser. Wir erwarteten ein solches zeitliches Nutzungsprofil, da die Studierenden parallel zahlreiche andere Kurse (Pharmakologie, HNO, klinische Chemie, Innere Medizin, Mikrobiologie/Hygiene, Anästhesie sowie Medizinethik) absolvierten.

Während der Aufwand der Erstellung einer eLearning-Plattform sowohl inhaltlich als auch technisch hoch ist, sehen wir dies als wertvolle Investition in Lehrveranstaltungen. Der größte Anteil der Arbeit wird nur einmalig geleistet, von einer verbesserten Lehre profitieren jedoch alle in Zukunft folgenden Lernenden. Dies beschränkt sich nicht nur auf die Lehre für Medizinstudierende. Auch in der Aus- bzw. Weiterbildung medizinischer Fachangestellter oder von Weiterbildungsassistenten sehen wir zukünftig einen großen und weiterhin wachsenden Stellenwert von didaktisch wertvoll gestaltetem eLearning [1]. Ein vorbildliches Beispiel hierfür ist der Start des DOG E‑Campus. In Zeiten von immer weiter verdichteter Arbeit und weniger Raum für Lehre können mit gutem eLearning durch Zeitmangel entstehende Defizite abgedämpft werden. Derzeit gibt es keine Daten, welche den Einsatz von eLearning für die Ausbildung im Gesundheitswesen als nachteilig gegenüber klassischen Formaten darstellen [4]. Einen Ersatz persönlicher Lehre und praktischer Anleitung stellt auch ein gut umgesetztes eLearning-Format selbstverständlich nicht dar.

Die kontinuierliche Pflege einer eLearning-Plattform sollte nicht vernachlässigt werden, um aktuelle inhaltliche und technische Aspekte abzubilden.

## Ausblick

Das Konzept der eingerichteten eLearning-Plattform hat sich bewährt, weshalb wir diese weiter pflegen und nutzen werden. Ein Ausbau mit zusätzlichen interaktiven Elementen ist geplant. Weiterhin ist eine entsprechende Plattform für den Untersuchungskurs der Augenheilkunde im 5. Semester geplant.

Die Einschränkungen für Präsenzlehre durch die SARS-nCoV-Pandemie des Sommersemesters 2020 wurden genutzt, um das Angebot unseres eLearnings mit Untersuchungsvideos, interaktiven Patientenfällen, Vorlesungs-Videopodcasts und weiteren multimedialen Elementen auszuweiten. Evaluationsdaten hierfür werden gesammelt und ausgewertet.

## Fazit für die Praxis

- Das vernetzende Konzept unserer eLearning-Plattform wurde positiv aufgenommen und lässt sich auch für andere Fächer und Ausbildungssituationen nutzen.
- Ein einmaliger Aufwand für Erstellung und eine maßvolle Menge an Pflegearbeit kann die Lehre dauerhaft verbessern.
- Die Kenntnis von Lernverhalten und realistische Erwartungen an den Nutzungsumfang durch die Lernenden können helfen, eLearning-Plattformen sinnvoll zu gestalten.
